# Whole exome sequencing identifies novel predisposing genes in neural tube defects

**DOI:** 10.1002/mgg3.467

**Published:** 2018-11-10

**Authors:** Philippe Lemay, Patrizia De Marco, Monica Traverso, Elisa Merello, Alexandre Dionne‐Laporte, Dan Spiegelman, Édouard Henrion, Ousmane Diallo, François Audibert, Jacques L. Michaud, Armando Cama, Guy A. Rouleau, Zoha Kibar, Valeria Capra

**Affiliations:** ^1^ CHU Sainte‐Justine Research Center University of Montréal Montréal Québec Canada; ^2^ IRCCS Istituto Giannina Gaslini Genoa Italy; ^3^ Montreal Neurological Institute McGill University Montréal Québec Canada; ^4^ Department of Obstetrics and Gynecology University of Montréal Montréal Québec Canada; ^5^ Department of Pediatrics University of Montréal Montréal Québec Canada; ^6^ Department of Neurosciences University of Montréal Montréal Québec Canada

**Keywords:** Neural tube defects (NTD), whole exome sequencing (WES), Molecular inversion probes (MIP) resequencing, candidate genes

## Abstract

**Background:**

Neural tube defects (NTD) are among the most common defects affecting 1:1000 births. They are caused by a failure of neural tube closure during development. Their clinical presentation is diverse and dependent on the site and severity of the original defect on the embryonic axis. The etiology of NTD is multifactorial involving environmental factors and genetic variants that remain largely unknown.

**Methods:**

We have conducted a whole exome sequencing (WES) study in five new NTD families and pooled the results with WES data from three NTD families and 43 trios that were previously investigated by our group. We analyzed the data using biased candidate gene and unbiased gene burden approaches.

**Results:**

We identified four novel loss‐of‐function variants in three genes, *MTHFR*,*DLC1,* and *ITGB1*, previously associated with NTD. Notably, *DLC1* carried two protein truncating variants in two independent cases. We also demonstrated an enrichment of variants in *MYO1E* involved in cytoskeletal remodeling. This enrichment reached borderline significance in a replication cohort supporting the association of this new candidate gene to NTD.

**Conclusion:**

These data provide some key insights into the pathogenic mechanisms of human NTD and demonstrate the power of next‐generation sequencing in deciphering the genetics of this complex trait.

## 1 INTRODUCTION

Neural tube defects (NTD) represent a group of congenital malformations that affect 1–2 individuals per 1,000 births. They are caused by incomplete neural tube closure during early development (De Marco, Merello, Mascelli, & Capra, 2006; Bassuk & Kibar, 2009). The most common forms of NTD are referred to as open NTD and include anencephaly, and myelomeningocele (MMC or spina bifida), which result from the failure of fusion in the cranial and spinal region of the neural tube, respectively. All infants with anencephaly are stillborn or die shortly after birth, whereas many infants with spina bifida now survive but with severe and lifelong physical and developmental disabilities. A number of skin‐covered (closed) NTD are categorized clinically depending on the presence (including lipomyeloschisis, lipomyelomeningocele, and meningocele) or absence of a subcutaneous mass (including dermal sinus and caudal agenesis) (Rossi et al., 2004). The variability of the phenotype can be attributed to temporal or spatial differences in the initial insult leading to NTD. The severity of these malformations ranges from asymptomatic to paralysis or prenatal death (Bassuk & Kibar, 2009).

Over the years, epidemiologic studies have been instrumental in elucidating the causes of NTD in humans. Overall, these studies have suggested that environmental and genetic factors have a joint role in the causation of NTD. Maternal diabetes has long been associated with NTD risk (Loeken, 2005). However, the most important epidemiological finding in NTD is the protective effect of peri‐conceptional intake of folic acid was shown to reduce the incidence of NTD by 50%–70% (MRC Vitamin Study Research Group, 1991). However, a large portion of NTD remains folate resistant demonstrating the need for novel preventive strategies. The vitamin‐like molecule inositol may offer a novel approach to preventing folic acid‐resistant NTD. Given the preventive effect of inositol in mice and the safety of this vitamin‐like molecule in a variety of human conditions, inositol as an adjunct therapy to folic acid for the prevention of folate‐resistant NTD is currently trialed (Greene et al., 2016).

Neural tube defects have a well‐established genetic component with a heritability of 60% (Bassuk & Kibar, 2009). While previous studies have identified over ~250 causative genes in mouse NTD segregating in a Mendelian fashion (Harris & Juriloff, 2007, 2010), the genetic etiology of the human disease remains largely unknown. Genomewide linkage studies have shown significant association with genomic regions on chromosomes 2, 7, and 10, but failed to identify causative genes (Stamm et al., 2008; Rampersaud et al., 2005). Candidate gene approaches have focused on folic acid pathway and on orthologues and/or homologues of mouse NTD genes. A few variants in folate‐related genes have been found to be significantly associated with an increased risk for NTD; however, they did not contribute substantially to the etiology of NTD and the burden of NTD in the population under study (Greene, Stanier, & Copp, 2009). Planar cell polarity (PCP) genes represent strong candidate genes for NTD as inferred from mouse models and have been extensively studied in human NTD cohorts. The fact that PCP proteins interact physically and functionally with ciliary proteins suggested the possibility that PCP signals may govern ciliogenesis, a process that is required for the correct development and patterning of cranial and spinal neural tube (Greene, Stanier, & Copp, 2009). Rare variants in genes of PCP pathway were associated with NTD in 1%–2% of patients analyzed (Greene et al., 2009). Most of these variants were missense and were detected in apparently healthy parents demonstrating an important role of variants of low penetrance in NTD etiology (Kibar et al., 2007; De Marco et al., 2014). The rise of powerful next‐generation sequencing (NGS) has brought immense advances to the field of genetics of both simple and complex traits. Therefore, our group has previously conducted whole exome sequencing (WES) in 43 trios affected with severe forms of NTDs (mainly MMC and anencephaly) and identified an important role for de novo variants in their etiology. We also previously used WES in three families each with two MMC cases as well as molecular inversion probe sequencing in a larger cohort, and we demonstrated a strong implication of *GRHL3* (Grainyhead Like Transcription Factor 3; OMIM *608317) in the etiology of MMC (Lemay et al., 2015; Lemay et al., 2017). In our previous WES studies, we aimed at investigating the role of highly deleterious variants, defined as frameshift, stop, or splicing variants, in the causation of open and severe NTD (mainly MMC). In this study, we conducted WES in five new families affected with both forms of open and closed NTDs and we reassessed the previously published cohorts consisting mainly of open MMC (Lemay et al., 2015; Lemay et al., 2017). WES data were analyzed using a biased candidate gene approach and an unbiased genetic burden approach.

## 2 METHODS

### 2.1 Cohort

**Figure 1:**
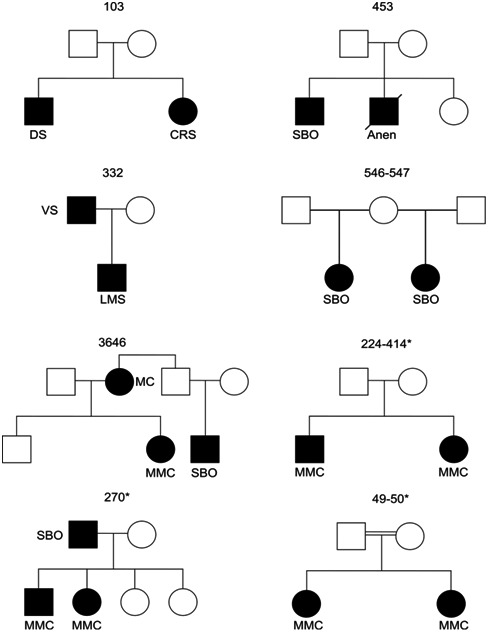
Representation of the eight neural tube defects affected families included in this study. The three families previously analyzed by our group are denoted by a star. Myelomeningocele (MMC), spina bifida occulta (SBO), vertebral schisis (VS), dermal sinus (DS), sacral agenesis (SA), anencephaly (Anen), myelocele (MC), and lypomyeloschisis (LMS)

Five new multiplex families were recruited for this study. NTD phenotypes recruited consisted of MMC, anencephaly, myelocystocele, spina bifida occulta, dermal sinus, lipomyeloschisis, caudal agenesis, and vertebral schisis. Families were recruited through the Istituto Giannina Gaslini in Genoa, Montreal Sainte‐Justine Hospital Spina Bifida Center and the 3D study of the Integrated Research Network in Perinatology of Quebec and Eastern Ontario. Three families each including two MMC cases and 43 trios previously published were reanalyzed using novel approaches as described below (Lemay et al., 2015; Lemay et al., 2017). All eight families are described in Figure 1. WES data from 188 nonfamilial controls were obtained through a collaboration with Guy A. Rouleau from McGill University. Written informed consent was obtained from all participating individuals.

### 2.2 Ethical compliance

The study was performed in accordance with the ethical guidelines for human subject research and was approved by the Local Institutional Review Board: IRCCS Istituto Giannina Gaslini (Protocol number: 213/2013) and CHU Ste‐Justine Hospital (Protocols’ numbers: 2598 and 2899).

### 2.3 WES and mutation analyses

Whole exome sequencing of the five new multiplex families was done as previously described in Lemay et al. (2015). This resulted in 96.51% of base pair being covered with at least 20 reads for the families. We analyzed both inherited and de novo mutations in all five new families and the previously investigated cohort of three families and 43 trios. This resulted in a cohort of 18 affected familial members and 43 affected trios probands. Candidate mutations were identified through two methods: candidate gene and genetic burden approaches. Basic filtering for all approaches removed synonymous, noncoding, and common (>1%) mutations. It kept high‐quality mutations (GQ = 99, forward mutation reads >3, reverse mutation reads >3, mutation frequency >0.15). Loss‐of‐function (LOF) mutations and missense mutations predicted to be probably damaging by Polyphen‐2 were prioritized for further analyses using a candidate gene and genetic burden approaches. The latter were prioritized to minimize background noise caused by nonbiologically active mutations (Adzhubei et al., 2010).

For the candidate gene approach, 221 genes classified as mouse NTD genes were obtained from Harris and Juriloff's reviews (Harris & Juriloff, 2007, 2010). A screen of 93 genes involved in the folate pathway from cellular transport to its involvement in thymidine formation and methylation based on the gene ontology database was next used to complete the list (Ashburner et al., 2000; The Gene Ontology Consortium, 2017). This resulted in a list of 314 genes for prioritization. Mutations previously identified by our group were removed. Mutations that were shared by all affected members of a family were prioritized.

The genetic burden analysis compared the number of mutations in each gene from the affected of five new families, 43 published trios (Lemay et al., 2015), and the three previously published families (Lemay et al., 2015) to the number in 188 ethnically matched nonfamilial controls. Genes with high mutation count due to misalignment problems were removed from analysis. Mutation rates were defined using the following formula: mutations/(cohort size × gene size × 2 alleles). The control cohort mutation rate for each gene was compared to the observed rate in our cohort using a two‐tailed Poisson test and corrected using a Bonferroni threshold of 0.05/20,389 genes = 2.45 × 10^−6^. The remaining genes were filtered based on biological plausibility.

### 2.4 MIP sequencing

Molecular inversion probes (MIPs) resequencing was used to investigate the novel NTD candidates identified by the gene burden approach. MIPs were designed using the scripts of O'Roak et al. (2012) available at http://krishna.gs.washington.edu/mip_pipeline/. The absence of SNPs in the MIPs arms was verified to insure unbiased capture. Eighty‐three MIPs were designed to cover the *MYO1E* (NM_004998.3) gene using the standard capture length of 112 bp. MIPs were tested for capture level, and poor performers were adjusted in the final mix. The sequencing reactions of the 83 MIPs in the 192 unrelated cases and 192 unrelated controls were run on one lane of Hiseq 2000. Data processing was done as described by Lemay et al. (2015). Mutations on resequenced controls were filtered for mutations with a frequency <1% and a probably damaging PolyPhen‐2 tag. Mutation rates were compared using a two‐tailed Poisson test.

## 3 RESULTS

### 3.1 Candidate gene approach

As a first approach, LOF mutations in genes related to the folate pathway or previously associated with NTD in mouse models were prioritized. This resulted in a list of 314 candidate NTD genes. Variants were first analyzed without segregation analysis in all 18 familial affected (from the five new families and three published families) and the 43 trios probands. After filtering out previously published mutations on *SHROOM3, PAX3,* and *GRHL3*, five new candidate LOF variants were identified: c.1683G>A (p.(Trp561Ter) in *MTHFR* (NM_005957.4) in Family 224–414, c.1066C>T (p.(Gln356Ter) in *DLC1* (NM_006094.4) in trio 25, c.121C>T (p.(Gln41Ter) in *DLC1* in Family 3646, c.2303_2304insA (p.(Lys768 fs) in *ITGB1* (NM_133376.2) in trio 260, and c.99_100insT (p.(Gly33fs) in *APAF1* (NM_181868.1) in Family 270 (Table 1). They were all absent from the ExAC, gnomAD databases. We next analyzed the segregation of these five new LOF variants in other family members. The *APAF1* variant was not shared by the other affected members of Family 270 reducing its candidacy and was hence removed from further analysis.

**Table 1:** Novel loss‐of‐function variants identified in Neural Tube Defects patients

Family ID	Chr	Position	Gene	cDNA change	Protein change	GnomAD	ExAc
224–414	1	11851333	*MTHFR*	c.1683G>A	(p.(Trp561Ter)	–	–
3646	8	13357460	*DLC1*	c.121C>T	(p.(Gln41Ter)	–	–
25	8	12957469	*DLC1*	c.1066C>T	(p.(Gln356Ter)	–	–
260	10	33197323	*ITGB1*	c.2303_2304insA	(p.(Lys768 fs)	–	–
270	12	99042236	*APAF1*	c.99_100insT	(p.(Gly33 fs)	–	–

Accession numbers: *MTHFR* (NM_005957.4); *DLC1* (NM_006094.4); *ITGB1* (NM_133376.2); *APAF1* (NM_181868.1).

**Figure 2:**
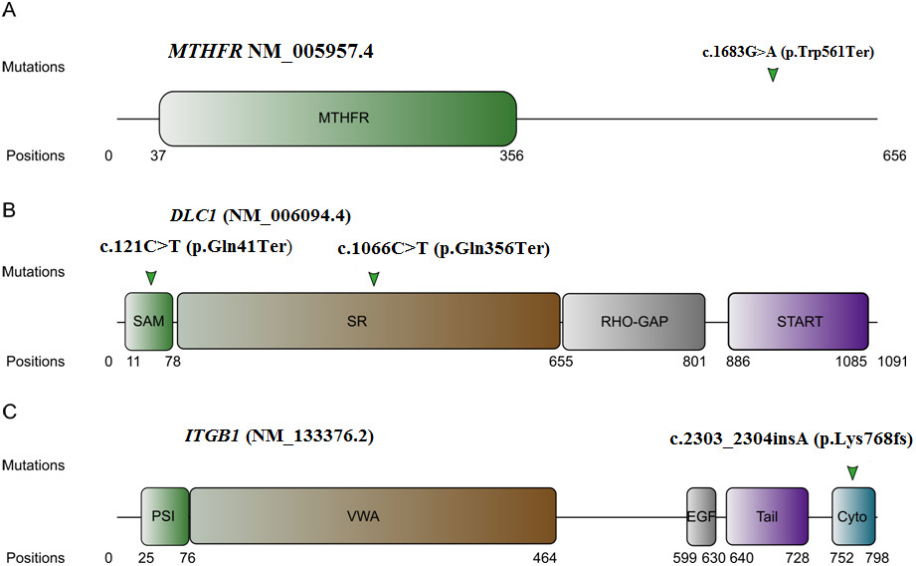
A schematic representation of MTHFR (a), DLC1 (b), and ITGB1 (c) indicating the position of the four novel loss‐of‐function variants identified in Neural Tube Defects patients

*MTHFR* codes for *methylenetetrahydrofolate reductase* that is involved in the transformation of 5‐10‐methyltetrahydrofolate to 5‐methyltetrahydrofolate, a cofactor of the homocysteine to methionine transition. The (p.(Trp561Ter) variant truncates 95 amino acids of the C‐terminus (Figure 2a). It was shared by the two sons affected with MMC but was transmitted by the unaffected mother. *Deleted in liver cancer 1* (*DLC1*) is tumor suppressor gene that codes for a RhoGTPase activating protein inactivated in many types of cancer. It has four functional domains: SAM, SR region, RHO‐GAP, and START (Figure 2b). The (p.(Gln356Ter) change truncates the last 299 aa of the SR region and the RHO‐GAP and START domains of DLC1 (Figure 2b). It was shared by the MMC proband and the unaffected mother. The (p.(Gln41Ter) variant in DLC1 truncates the last 37aa of the SAM domain and all three other functional domains of DLC1. It was shared by the MMC affected daughter, the myelocele affected mother and cousin, and the unaffected brother. *Integrin beta 1* (*ITGB1*) codes for an integrin involved in linking the actin cytoskeleton to the extracellular matrix (Ohyama, Kawano, & Kawamura, 1997). ITGB1 has five functional domains: the PSI domain, the integrin beta subunit VWA domain, the EGF‐like domain, integrin beta subunit tail domain, and the cytoplasmic domain. The (p.(Lys768fs) variant causes a premature stop codon mutation truncating more than half of the cytoplasmic domain and the last 30aa of the protein. It was shared between the MMC proband and the unaffected mother. Missense variants with probably damaging PolyPhen‐2 scores were also investigated in NTD candidate genes to identify additional variants that are shared by affected family members and that would affect the penetrance of the NTD phenotype. We identified 63 probably damaging variants in 36 families or trios (Supporting Information Table S1). Eight variants were novel and one individual (pt 53) was carrier of two never reported damaging variants, the *FREM2* (NM_207361.5) c.413A>C (p.(Tyr138Ser) and the *TCN2* (NM_001184726.1) c.1189C>G (p.(Leu397Val). Intriguingly, three patients were carriers of very rare missense variants in genes like *IFT172* and *C2CD3* encoding for proteins necessary for ciliary assembly and maintenance. None of these candidates segregated in a compound heterozygous transmission with one of the previously identified LOF variants or other missense changes in this cohort.

### 3.2 Genetic burden approach

**Figure 3:**
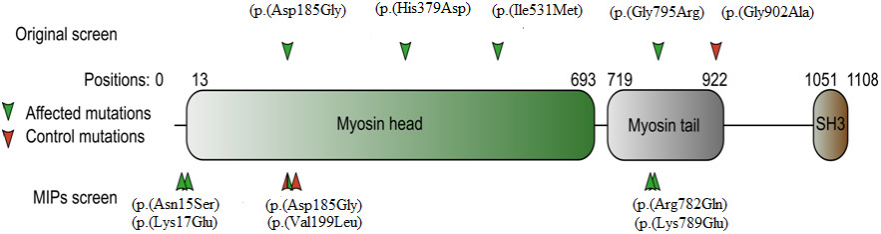
Graphical representation of the MYO1E protein indicating the position of Neural Tube Defects (NTD)‐associated variants. Variants identified by whole exome sequencing or by MIP sequencing are on top and bottom sides of the protein, respectively. Variants identified in NTD patients are indicated by green arrows and those in controls by red arrows

Variants with low frequency (MAF <1%), LOF variants, and probably damaging PolyPhen‐2 tag were identified to investigate a genetic burden in our cohort. Identical filtration parameters were applied to a cohort of 188 ethnically matched nonfamilial controls. Significant enrichment under the 2.45 × 10^−6^ Bonferroni threshold was found in *Dysferlin* (*DYSF*) and *MYO1E* (P values = 1.228 × 10^−6^). DYSF is a 230 kDa protein involved in sarcolemma repair and Ca^2+^ reactivity (Bansal et al., 2003). It is expressed throughout development, but remains mostly involved in muscle formation and hence was not considerate as a biologically plausible gene for NTD (Anderson et al., 1999). *MYO1E* is an unconventional nonmuscle class I myosin with a motor‐head domain that binds ATP and F‐actin, a calmodulin‐binding neck domain and a tail domain (Mele et al., 2011). It had six variants in the 61 affected and only one in the 188 controls (Figure 3 and Table 2). Given the crucial importance of actin remodeling in neural tube formation, MYO1E was considered a strong candidate for NTD and was further investigated by MIP resequencing of 192 unrelated cases and 192 unrelated controls using MIPs. Identified variants were filtered following the same criteria as before. Five variants were identified in this gene in the affected cohort and two in the unaffected (Figure 3 and Table 2). Using a two‐tailed Poisson test, these data suggest a borderline significant *p* value of 0.05265 in the replication cohort.

**Table 2:** Variants identified in the *MYO1E* [Link] gene in neural tube defects (NTD) patients and controls

Family ID	Type	Chr	Position	cDNA change	Protein change	rs number	EVS frequency	gnomAD frequency	PolyPhen score
Whole exome sequencing cohort									
201	NTD	15	59519746	c.554A>G	(p.(Asp185Gly)	rs141565214	0.001157	0.002103	0.99
265/553	NTD	15	59506892	c.1135C>G	(p.(His379Asp)	rs150983259	0.001543	0.001320	0.982
28/552	NTD	15	59497622	c.1593C>G	(p.(Ile531Met)	rs140447165	0.006480	0.007179	0.988
389	NTD	15	59464193	c.2383G>A	(p.(Gly795Arg)	rs180951130	0.000077	0.0005989	0.997
574	Control	15	59453352	c.2705G>C	(p.(Gly902Ala)	–	0	–	0.993
MIPS cohort									
10179	NTD	15	59564608	c.A2365G	(p.(Lys789Glu)	rs542281660	0	1.840 e‐5	0.925
10194	NTD	15	59564603	c.G2345A	(p.(Arg782Gln)	–	0	–	0.93
10278	NTD	15	59519746	c.A554G	(p.(Asp185Gly)	rs141565214	0.001157407	0.002103	0.866
10190	NTD	15	59464231	c.A49G	(p.(Lys17Glu)	–	0	1.625 e‐5	0.85
10322	NTD	15	59464211	c.A44G	(p.(Asn15Ser)	–	0	2.031 e‐5	0.901
20871	Control	15	59519705	c.G595T	(p.(Val199Leu)	–	0	8.122 e‐6	0.848
20785	Control	15	59519746	c.A554G	(p.(Asp185Gly)	rs141565214	0.001157407	0.002103	0.866

*MYOE* accession number (NM_004998.3).

## 4 DISCUSSION

In this study, we conducted WES in five new NTD families affected with both forms of open and closed NTD. We next pooled these data with WES data from three previously published NTD families and 43 NTD trios and analyzed the data using biased and unbiased approaches. Our strategy allowed us to identify four novel LOF variants in three genes previously associated with NTD (*MTHFR*,* DLC1,* and *ITGB1*) and notably a significant mutation burden in a novel gene *MYO1E*. A replication MIP resequencing study in a larger NTD cohort identified an enrichment in this novel gene that approached statistical significance.

*MTHFR* is one of the most studied genes in human NTD with more than 10 known mutations shown to modify its enzymatic activity (Leclerc, Sibani, & Rozen, 2013). The (p.(Trp561Ter) variant identified in NTD in this study truncates 95 amino acids of the C‐terminus which has been associated with protein stability (Shan, Wang, Hoffmaster, & Kruger, 1999). *DLC1* was previously associated with defect in neural tube closure through a gene trap mouse model (Sabbir et al., 2010). It can down‐regulate RHO that is a known effector of the PCP pathway and an important factor in the localization of actin filaments during apical constriction. The SAM domain is involved in protein–protein interaction with EF1A1 and has an auto‐inhibitory effect on the DLC1 protein (Kim et al., 2008). The SR region interacts with the SH2 domains of TENSIN1 and CTEN targeting DLC1 to the membrane where it can enact its RHO‐GAP activity (Qian et al., 2007). The RHO‐GAP domain hydrolyze RHOa‐GTP to RHOa‐GDP that is a key process in cell proliferation, cell morphology, and cell migration (Liao, Si, deVere White, & Lo, 2007; Kim et al., 2007). The START domain function remains unclear, but may be involved in DLC1 inhibition of actin stress fiber formation (Sabbir et al., 2010). *DLC1* carried two LOF variants, (p.(Gln41Ter) and (p.(Gln356Ter), in two NTD families: (p.(Gln41Ter) that truncates all four functional domains and (p.(Gln356Ter) that truncates three making them highly deleterious. Since both variants truncate the RHO‐GAP domain, it is highly probable that the resulting protein will be inactive making them highly pathogenic nucleotide changes. *ITGB1* has been associated with NTD through a knock‐in mouse showing an exencephaly and spina bifida (Ohyama, Kawano & Kawamura, 1997). This seems to be linked with the reduced migratory potential of cells which could lead to reduced cellular rearrangements, a key process involved in the neural tube formation (Baudoin et al., 1998). The (p.(Lys768 fs) variants would truncate the cytoplasmic domain of the ITGB1 protein that is essential signal transduction between the cytoskeleton and the extracellular matrix (Ohyama, Kawano & Kawamura, 1997). All four LOF variants detected in these three NTD candidate genes were detected in unaffected members suggesting incomplete penetrance (Supporting Information Figure S1). We did not identify any other probably damaging variant that was shared by the affected, but absent from the unaffected members that segregated with the LOF variants. This suggests the presence of other potentially synergistic variants that were not prioritized in our approach (e.g. variants that have an MAF >1% or predicted to be benign) and/or environmental factors that modulated the penetrance of the NTD phenotype in these families.

Using an unbiased gene burden analysis, we were able to identify a novel candidate gene *MYO1E* that was significantly enriched for variants related to NTD. While the replication cohort did not reach the significance level, the borderline *p* value still represented this gene an excellent candidate for NTD. Genetic variants on this gene lead to focal segmental glomerulosclerosis characterized by a thickening and disorganization of the glomerular basement membrane of podocytes (Mele et al., 2011). *MYO1E* is expressed in fetal brain and one could hypothesize that predisposing variants in this gene could affect the extensive morphological changes that are largely actin/myosin based (Skoglund, Rolo, Chen, Gumbiner, & Keller, 2008). and that are essential for the processes of convergent extension and apical constriction during neural tube formation. Additional functional studies in cell‐based and animal models are needed to further investigate the role of this gene in the pathogenesis of NTD.

In conclusion, we have provided in this study and in our two previous studies a proof of principle for the power of NGS in deciphering the complex genetics of NTD. Despite the limitations of our cohort small sample size and using a combination of biased and unbiased analytical approaches, we were able to (a) identify de novo variants that may play a role in severe forms of NTD (MMC and anencephaly) (Lemay et al., 2015); (b) implicate *GRHL3* in the etiology of human NTD (Lemay et al., 2017); (c) identify LOF variants in orthologues of mouse NTD genes, including *SHROOM3*,* DLC1,* and *ITGB1*, in human NTD (the current study); and (d) identify *MYO1E* as a novel potential NTD candidate gene (current study). Using similar approaches in larger NTD cohorts will be instrumental in deciphering the complex genetics of NTD and will help develop personalized genetic counseling strategies in affected families.

## CONFLICT OF INTEREST

The authors report no conflict of interests.
