# Supplementary material for: Whole exome sequencing identifies novel predisposing genes in neural tube defects
Source: Mol Genet Genomic Med. 2018 Nov 10;7(1):e00467. doi: 10.1002/mgg3.467 (PMC6382446; doi:10.1002/mgg3.467)

# **Supplementary material**

# **Whole exome sequencing identifies novel predisposing genes in neural tube defects**

Philippe Lemay^1,^†, Patrizia De Marco^2,^†*, Monica Traverso^2^, Elisa Merello^2^, Alexandre Dionne-Laporte^3^, Dan Spiegelman^3^, Édouard Henrion^3^, Ousmane Diallo^3^, François Audibert^1,4^, Jacques L. Michaud^1,5^, Guy A. Rouleau^3^, Zoha Kibar ^1,6^, and Valeria Capra^2^

^1^ CHU Sainte-Justine Research Center, University of Montréal, 3175 Cote-Sainte-Catherine, Montréal,  QC H3T 1C5 Québec Canada;

^2^ Istituto Giannina Gaslini, v. G. Gaslini, 5 16147, Genoa, Italy;

^3^Montreal Neurological Institute, McGill University, 1001 Décarie Boulevard, Montréal, QC H4A 3J1 Québec Canada:

^4^Department of Obstetrics and Gynecology, University of Montréal, 1001 Décarie Boulevard, Montréal, QC H4A 3J1Québec Canada;

^5^Department of Pediatrics, University of Montréal, 1001 Décarie Boulevard, Montréal, QC H4A 3J1 Québec Canada:

^6^Department of Neurosciences, University of Montréal, 1001 Décarie Boulevard, Montréal, QC H4A 3J1 Québec Canada.

*Corresponding author: Patrizia De Marco, Laboratorio Neurogenetica e Neuroscienze- Istituto Giannina Gaslini-Genova, Italy; Phone: 39-010-56363308; Fax:39-010-3993159; E-mail: [patriziademarco@gaslini.org](mailto:patriziademarco@gaslini.org)

ORCID number: 0000-0003-3915-7

|  | Supp. Table 1 | | | | | |  |  |  |  |
| --- | --- | --- | --- | --- | --- | --- | --- | --- | --- | --- |
| Family | Chr | Position | Gene | NM number | cDNA change | Protein change | | EVS frequency | GnomAD frequency | Polyphen-2 score |
| 6 | 8 | 144873405 | *SCRIB* | NM_182706 | c.4896T>A | p.Glu1632Asp | | 0.000704 | 0.0005449 | 0.877 |
| 11 | 4 | 77676155 | *SHROOM3* | NM_020859 | c.4519G>A | p.Glu1507Lys | | 0.001153 | 0.001535 | 0.903 |
| 20 | 1 | 22160001 | *HSPG2* | NM_005529 | c.10937G>A | p.Arg3646His | | 0.003383 | 0.003213 | 1.0 |
| 20 | 6 | 15496729 | *JARID2* | NM_004973 | c.1273C>T | p.Arg425Trp | | 0 | 2.565 e -5 | 0.977 |
| 25 | 12 | 125298858 | *SCARB1* | NM_005505 | c.520C>T | p.Arg174Cys | | 0 | 4.329 e - 5 | 0.999 |
| 28 | 7 | 75614157 | *POR* | NM_000941 | c.1129T>A | p.Tyr377Asn | | 0 | 3.236 e -5 | 1.0 |
| 31 | 1 | 15832495 | *CASP9* | NM_032996 | c.461A>C | p.His154Pro | | 0.001845 | 0 | 1.0 |
| 31 | 2 | 233272029 | *ALPPL2* | NM_031313 | c.218T>C | p.Ile73Thr | | 0.001768 | 0.002314 | 0.914 |
| 31 | **8** | **144887523** | ***SCRIB*** | **NM_182706** | **c.2429G>A** | **p.Arg810Gln** | | **0** | **0** | **0.997** |
| 31 | 9 | 77357506 | *TRPM6* | NM_017662 | c.5171C>T | p.Thr1724Ile | | 0.001307 | 0.001029 | 0.97 |
| 31 | 17 | 54671835 | *NOG* | NM_005450 | c.251C>A | p.Pro84His | | 0.000697 | 0.001266 | 0.999 |
| 32 | **7** | **74159167** | ***GTF2I*** | **NM_033001** | **c.1758G>C** | **p.Leu586Phe** | | **0** | **0** | **0.958** |
| 32 | 11 | 59622246 | *TCN1* | NM_001062 | c.1000A>G | p.Ile334Val | | 0.003233 | 0.003070 | 0.931 |
| 53 | **13** | **39261894** | ***FREM2*** | **NM_207361** | **c.413A>C** | **p.Tyr138Ser** | | **0** | **0** | **0.999** |
| 53 | **22** | **31022494** | ***TCN2*** | **NM_001184726** | **c.1189C>G** | **p.Leu397Val** | | **0** | **0** | **0.951** |
| 67 | 20 | 60885119 | *LAMA5* | NM_005560 | c.10753G>T | p.Asp3585Tyr | | 0.001543 | 0.001450 | 0.946 |
| 67 | 22 | 46777777 | *CELSR1* | NM_014246 | c.7054C>T | p.Pro2352Ser | | 0.000155 | 0.0001189 | 1.0 |
| 79 | 6 | 139694603 | *CITED2* | NM_006079 | c.479A>T | p.His160Lys | | 0.002076 | 0.001568 | 0.999 |
| 79 | 16 | 2131695 | *TSC2* | NM_001114382 | c.3710C>T | p.Ala1237Val | | 0 | 2.851 e -5 | 0.96 |
| 122 | 8 | 144893352 | *SCRIB* | NM_182706 | c.1070C>T | p.Thr357Met | | 0 | 6.466 e-5 | 0.997 |
| 125 | 16 | 2112558 | *TSC2* | NM_001114382 | c.1318G>A | p.Gly440Ser | | 0.001154 | 0.0007397 | 0.981 |
| 134 | **2** | **233246399** | ***ALPP*** | **NM_001632** | **c.1502C>G** | **p.Pro501Arg** | | **0** | **0** | **0.987** |
| 134 | 22 | 41545151 | *EP300* | NM_001429 | c.2351C>T | p.Pro784Leu | | 0.000154 | 0.0001299 | 0.989 |
| 138 | 2 | 27681015 | *IFT172* | NM_015662 | c.2953G>A | p.Gly985Ser | | 0.000077 | 0.0001056 | 0.86 |
| 138 | 5 | 52397199 | *MOCS2* | NM_004531 | c.367C>T | p.His123Tyr | | 0.004075 | 0.003585 | 1 |
| 138 | 11 | 64575091 | *MEN1* | NM_130804 | c.731T>C | p.Met244Thr | | 0 | 4.062 e -6 | 0.999 |
| 138 | 11 | 73795965 | *C2CD3* | NM_015531 | c.3961G>A | p.Val1321Ile | | 0.000539 | 0.0001155 | 0.92 |
| 138 | 16 | 86602272 | *FOXC2* | NM_005251 | c.1331A>G | p.Gln444Arg | | 0.001002 | 0.001136 | 0.98 |
| 138 | 17 | 48761052 | *ABCC3* | NM_003786 | c.3889C>T | p.Arg1297Cys | | 0.000077 | 2.918 e -5 | 1 |
| 191 | 1 | 24657999 | *GRHL3* | NM_198174 | c.101C>T | p.Thr34Met | | 0.000077 | 5.686 e -5 | 0.976 |
| 191 | 2 | 240005859 | *HDAC4* | NM_006037 | c.G2509A | p.Val837Met | | 0 | 2.909 e -5 | 0.972 |
| 191 | 13 | 39433629 | *FREM2* | NM_207361 | c.C7421A | p.Pro2474His | | 0 | 1.627 e -5 | 0.899 |
| 201 | 11 | 89405082 | *FOLH1B* | NM_153696 | c.209G>A | p.Arg70Gln | | 0.002617 | 0 | 1 |
| 202 | 1 | 1559159 | *MIB2* | NM_080875 | c.598C>T | p.Arg200Trp | | 0.000082 | 3.237 e -5 | 0.999 |
| 215 | **11** | **59604674** | ***GIF*** | **NM_005142** | **c.844G>A** | **p.Asp282Asn** | | **0** | **0** | **0.986** |
| 231 | 17 | 41226488 | *BRCA1* | NM_007300 | c.4598G>T | p.Ser1533Ile | | 0.002768 | 0.002324 | 0.975 |
| 258 | 14 | 31592235 | *HECTD1* | NM_015382 | c.5000G>A | p.Arg1667His | | 0.000081 | 4.706 e - 5 | 0.943 |
| 260 | 6 | 39877666 | *MOCS1* | NM_005943 | c.1015C>T | p.Arg339Trp | | 0.000231 | 0.0004481 | 1 |
| 260 | 22 | 46929838 | *CELSR1* | NM_014246 | c.3230C>T | p.Thr1077Met | | 0.001538 | 0.001125 | 1.0 |
| 263 | 3 | 49455293 | *AMT* | NM_001164712 | c.991C>T | p.Arg331Trp | | 0 | 1.806 e -5 | 1 |
| 269 | 4 | 169825056 | *PALLD* | NM_016081 | c.2570A>G | p.Gln857Arg | | 0 | 2.846 e -5 | 0.998 |
| 269 | 17 | 29585373 | *NF1* | NM_001042492 | c.4185G>T | p.Gln1395His | | 0 | 3.252 e -5 | 0.978 |
| 389 | 20 | 60900593 | *LAMA5* | NM_005560 | c.5308C>T | p.Arg1770Trp | | 0.000154 | 0.0002226 | 1.0 |
| 394 | **2** | **223096883** | ***PAX3*** | **NM_181461** | **c.706C>G** | **p.Arg236Gly** | | **0** | **0** | **0.998** |
| 402 | 1 | 160389002 | *VANGL2* | NM_020335 | c.403C>T | p.Arg135Trp | | 0 | 7.384 e -5 | 0.998 |
| 402 | 20 | 60901932 | *LAMA5* | NM_005560 | c.5203G>A | p.Val1735Met | | 0.006689 | 0.005683 | 0.977 |
| 453 | 19 | 11097098 | *SMARCA4* | NM_003072 | c.589C>TT | p.Pro197Ser | | 0.000928 | 0.003030 | 0.992 |
| 530 | 9 | 86912178 | *SLC28A3* | NM_022127 | c.819T>A | p.Phe273Leu | | 0 | 4.082 e -6 | 0.98 |
| 548 | 1 | 24668728 | *GRHL3* | NM_198174 | c.1171C>T | p.Arg391Cys | | 0 | 4.096 e -6 | 1.0 |
| 548 | 9 | 77411729 | *TRPM6* | NM_017662 | c.2319G>C | p.Gln773His | | 0.002460 | 0.001706 | 0.999 |
| 548 | 20 | 60893637 | *LAMA5* | NM_005560 | c.7112G>A | p.Arg2371His | | 0.001628 | 0.001356 | 0.998 |
| 550 | 20 | 50405627 | *SALL4* | NM_020436 | c.2515G>T | p.Val839Phe | | 0.000077 | 1.805 e -5 | 0.889 |
| 551 | 1 | 204518499 | *MDM4* | NM_002393 | c.1162C>G | p.Pro388Ala | | 0.000461 | 0.0006285 | 0.998 |
| 551 | 6 | 86197163 | *NT5E* | NM_002526 | c.1060C>T | p.Arg354Cys | | 0.000154 | 0.0001459 | 1 |
| 552 | **6** | **166580902** | ***T*** | **NM_003181** | **c.178A>G** | **p.Asn60Asp** | | **0** | **0** | **0.999** |
| 552 | 8 | 144895820 | *SCRIB* | NM_182706 | c.433G>A | p.Gly145Arg | | 0.000769 | 0.0007082 | 0.995 |
| 553 | 20 | 60893538 | *LAMA5* | NM_005560 | c.7211G>A | p.Arg404His | | 0.000619 | 0.0007010 | 0.951 |
| QKV | 3 | 125879755 | *ALDH1L1* | NM_012190 | c.68G>A | p.Gly23Asp | | 0.004306 | 0.004278 | 0.999 |
| QKV | 6 | 139694603 | *CITED2* | NM_006079 | c.479A>T | p.His160Leu | | 0.002076 | 0.001568 | 0.999 |
| QKV | 11 | 73801927 | *C2CD3* | NM_015531 | c.3572T>G | p.Val1191Gly | | 0 | 2.846 e -5 | 0.881 |
| QKV | 19 | 11488877 | *EPOR* | NM_000121 | c.1310G>A | p.Arg437His | | 0.002230 | 0.001162 | 0.932 |
| KKS | 4 | 169846140 | *PALLD* | NM_016081 | c.3218G>A | p.Gly1073Glu | | 0 | 6.514 e -5 | 0.986 |
| YPT | 17 | 48158680 | *ITGA3* | NM_005501 | c.2827A>G | p.Arg943Gly | | 0.007536 | 0.007063 | 0.939 |
| Novel variants in bold characters | | | | | | | | | | |


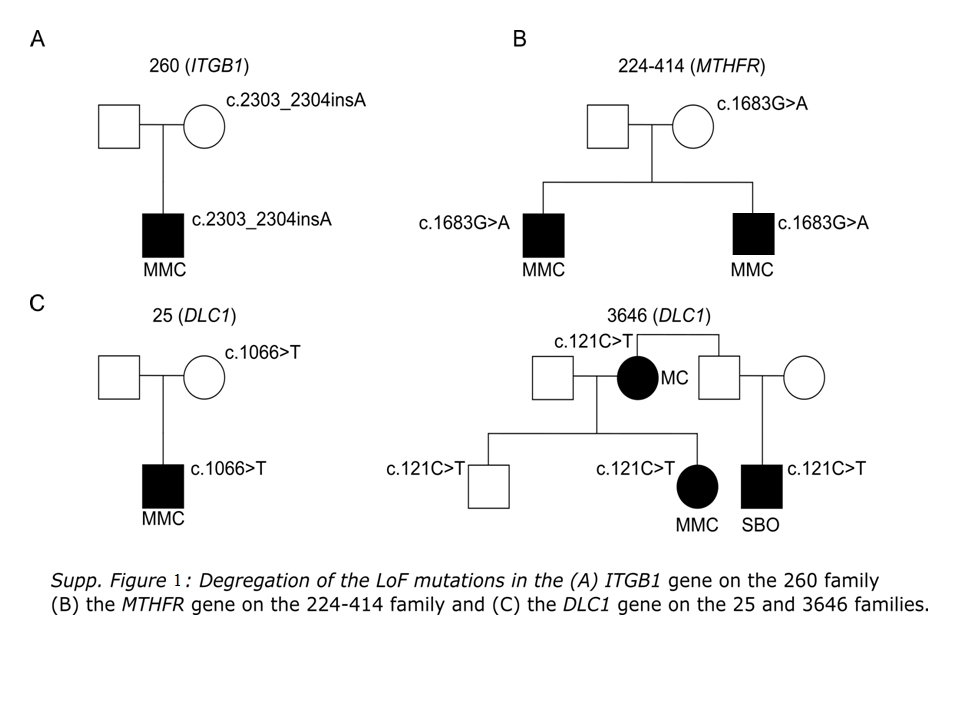

Supplement: Supplementary file 1 [file MGG3-7-na-s001.docx]
